# Symptom network analysis of insomnia-depression-anxiety-stigma in tuberculosis patients

**DOI:** 10.3389/fpsyt.2024.1513524

**Published:** 2025-01-23

**Authors:** Xiangmin Liu, Xue Qiu, Huizhen Lan, LiuYue Diao, Wei Huang, Yan Wen, Mei Feng, Xiangdong Tang

**Affiliations:** ^1^ Department of Pulmonary and Critical Care Medicine, West China Hospital, Sichuan University/West China School of Nursing, Sichuan University, Chengdu, China; ^2^ Department of Intensive Care Unit, The Fourth People Hospital of Nanning, Nanning, China; ^3^ Chengdu Center for Disease Control and Prevention, Chengdu, China; ^4^ Nursing Key Laboratory of Sichuan Province, Chengdu, China; ^5^ Mental Health Center/Neurobiology Monitoring Center, West China Hospital, Chengdu, China

**Keywords:** tuberculosis, insomnia, depression, anxiety, stigma, symptom network analysis

## Abstract

**Background:**

Insomnia, depression, anxiety, and stigma are prevalent and often coexist in patients with Tuberculosis (TB), potentially exacerbating one another. However, the complex intrinsic associations among these four disorders remain unclear, particularly concerning the role of stigma in relation to the other disorders.

**Methods:**

A cross-sectional study was conducted at West China Hospital and the Fourth People’s Hospital of Guangxi from November 2023 to June 2024. The levels of insomnia, depression, anxiety, and stigma among TB patients were assessed using the Pittsburgh Sleep Quality Index (PSQI), Patient Health Questionnaire-9 (PHQ-9), Generalized Anxiety Disorder-7 (GAD-7), and the TB-Related Stigma Scale (TRSS). Network analysis was used to identify the central and bridge symptoms and explore the role of stigma within the insomnia-depression-anxiety-stigma network.

**Results:**

PHQ1 (anhedonia), GAD1 (nervousness), GAD5 (restlessness), and PHQ3 (sleep problems) are central to the network. Bridge symptoms, including PHQ3 (sleep problems), PSQI5 (sleep disturbances), and GAD5 (restlessness) link the depression, insomnia, and anxiety communities. TRSS1 (family’s negative perception) of the stigma community exhibited the highest betweenness and second highest bridge betweenness in the network, highlighting the mediating role of family support across insomnia and psychological symptoms. Additionally, the global strength invariance test indicates that gender, age and education level do not significantly impact the network structure.

**Conclusion:**

Depression (anhedonia and sleep problems) and anxiety (nervousness and restlessness) are the primary concerns requiring intervention in TB patients. In addition, sleep problems act as a bridge in the overall network. Stigma, particularly negative perceptions from family, may play a crucial mediating role in sustaining the entire symptom network. Consequently, these symptoms could represent potential targets for intervention.

## 1 Introduction

As a chronic infectious disease, tuberculosis (TB) causes a significant public health threat and imposes a substantial medical burden globally (1). In 2023, TB caused 1.25 million deaths, likely making it the leading cause of death from a single infectious agent again, after being surpassed by COVID-19 for three years (2). China accounts for the third largest burden of TB in the world, representing 7.1% (approximately 748,000 cases) of global incidence, particularly in western China (2). Due to prolonged treatment, drug side effects, and lack of social support, TB patients are prone to sleep and mental problems such as insomnia, anxiety, and depression, with morbidity rates even exceeding 50% (1, 3), 30% (4), and 40% (5), respectively. Comorbidity of TB and these disorders can worsen disease progression by weakening immunity and reducing medication adherence, ultimately leading to decreased treatment success, increased mortality, and heightened transmission (6, 7). Therefore, it is essential to identify their intrinsic association and develop effective interventions accordingly.

Stigma is considered to be a major social stressor significantly affecting sleep and mental health. Approximately 42% to 82% of TB patients experience stigma of varying severity, manifesting in several detrimental ways, including avoidance by others, difficulties in accessing services and employment, as well as being insulted, marginalized, and rejected in interpersonal interactions (8). In the context of chronic stigmatization, people with TB may exhibit isolation, multiple worries, poor interpersonal sharing, and even hopelessness (9), all of which are classic symptoms of depression and anxiety. Additionally, stigma plays a crucial role in exacerbating sleep problems in TB patients. According to our previous study, TB-related stigma can significantly increase the risk of insomnia, with an odds ratio (OR) of 1.161 (1). To summarize, TB-related insomnia, anxiety, depression, and stigma are inextricably linked, resembling a complex system of interactions. Nevertheless, much of the current research (10–12) on these interconnected factors in TB patients is compartmentalized and fails to consider the overall interaction effects of all four conditions. Furthermore, each of these disorders—insomnia, anxiety, depression, and stigma—has distinct measurement dimensions, and the associations or interactions among these conditions across different dimensions in TB patients are still not fully understood.

Network analysis employs a symptom-oriented approach that computes indices for each node, such as centrality and predictability, reflecting the importance and controllability of a node within the network (13). Based on the nodes (symptoms), communities (symptom groups), and edges (associations) in the network, as well as the visualization model constructed using the graphical Lasso algorithm, researchers can perceive and understand the internal and external associations between different types of psychiatric disorders in patients (14). Of note, network analysis effectively considers both the overall level of disorders and the level of symptoms under dimensional segmentation, offering a reliable and explicit method to explore the complex interactions in mental disorders (15). Leveraging these network associations to identify the central and bridge symptoms provides essential support for physicians in treating symptoms and comorbidities in clinical practice (16). Earlier studies have shown that addressing the severity of bridge symptoms in dynamic psychopathology models may increase the effectiveness of interventions (17). Therefore, we investigated the complex intrinsic associations between insomnia, anxiety, depression, and stigma in TB patients through symptom network analysis. Through these efforts, we want to provide more effective and precise interventions to enhance sleep quality and mental health in individuals affected by TB.

## 2 Methods

### 2.1 Participants and procedure

Eligible patients at West China Hospital and the Fourth People’s Hospital of Guangxi were selected through convenience sampling from November 2023 to June 2024. Clinical data, including demographic information, sleep quality, depression, anxiety, and TB-related stigma were gathered through an electronic structured questionnaire on the day of admission. The Biomedical Research Ethics Committee of West China Hospital, Sichuan University approved the study.

#### 2.1.1 Inclusion criteria

1) Patients with diagnosed TB, following the National TB Program guidelines (18, 19); 2) Patients aged 14 years and above; 3) Patients who could complete the questionnaire with their communication and cognitive skills; and 4) Patients who provided informed consent to participate in this study.

#### 2.1.2 Exclusion criteria

1) Patients with severe physical disorders; 2) Patients with confirmed psychiatric disorders before TB was diagnosed; 3) Patients suffering from other infectious diseases, such as acquired immune deficiency syndrome (AIDS) or syphilis; 4) Special populations such as pregnant women; 5) Those experiencing critical incidents affecting sleep or mental health, such as severe trauma or bereavement.

### 2.2 Measures

#### 2.2.1 Insomnia

Was measured by the Pittsburgh Sleep Quality Index (PSQI), a widely used self-report questionnaire that assesses sleep quality and disturbances over one month. Nineteen individual items generate seven “component” scores: subjective sleep quality, sleep latency, sleep duration, habitual sleep efficiency, sleep disturbances, use of sleeping medication, and daytime dysfunction. Scores for these seven components are added to obtain a global score ranging from 0 to 21, and PSQI>10 indicates moderate-to-severe insomnia.

#### 2.2.2 Depression and anxiety

Were assessed using the Patient Health Questionnaire-9 (PHQ-9) (20) and Generalized Anxiety Disorder-7 (GAD-7), both of which are grounded in the Diagnostic and Statistical Manual of Mental Disorders, 4th Editions (DSM-IV) diagnostic criteria for measuring probable depression or anxiety over the past two weeks. Each item on the PHQ-9 and GAD-7 is rated on a 4-point Likert scale, ranging from 0 (not at all) to 3 (nearly every day). To quantify clinically significant depression and anxiety, we used a cut-off score of 10 or higher on the PHQ-9 and GAD-7.

#### 2.2.3 Stigma

Was assessed using the TB-related Stigma Scale (TRSS), which has demonstrated its suitability for measuring stigma among Chinese TB patients (21). This scale comprises nine items categorized into three subscales: Negative Experience, Emotional Reactions, and Coping Style. Responses are recorded on a 4-point Likert scale, ranging from ‘strongly disagree’ (0) to ‘strongly agree’ (3), with higher scores indicating a greater level of stigma.Chinese versions of all the above scales have been validated for clinical use, with excellent Cronbach’s alpha scores of 0.851–0.924 in this study. The abbreviations, content, and description of all scale items are shown in **Supplementary Table S1**, **Appendix A**.

### 2.3 Network analysis

#### 2.3.1 Network estimation

The Graphical Gaussian Model (GGM) uses a network to represent the relationships between variables in a multivariate Gaussian distribution. A network consists of nodes and edges. Nodes represent observed variables (e.g. attitudes and behaviors), and edges are associations between nodes. Specifically, we used the R (Version 4.2.3) package *qgraph* to estimate and plot the undirected cross-sectional Glasso network.

#### 2.3.2 Central and bridge symptoms

Centrality metrics are a set of metrics that can reflect the importance of a node in a network, including Strength Centrality, Closeness Centrality, Betweenness Centrality, and Expected Influence (EI). When the network contains both positive and negative edges, traditional centrality metrics such as Strength Centrality may not accurately predict the influence of nodes on the network (22). Therefore, we use the EI to determine the importance of nodes in this network analysis.

Comorbidity in a network analyzes connections among nodes to identify an individual’s co-occurrence of multiple diseases or medical conditions (15). The comorbidity of a network in which the nodes belong to different communities (e.g., anxiety community and depression community), linked by paths called bridges, can be explored via a bridge network. Bridge metrics can reflect the degree of association of nodes in a network with other communities, and further reveal the interactions between different communities. The bridge metrics include Bridge Strength Centrality, Bridge Closeness Centrality, Bridge Betweenness Centrality, and Bridge Expected Influence (BEI). Considering both positive and negative edges, this study uses the BEI of different nodes to examine network comorbidity. Nodes with high BEI are most likely to activate or be activated by nearby community nodes. Bridge nodes, having the highest BEI, should be targeted for intervention, as suppressing them may alleviate other symptoms.

#### 2.3.3 Accuracy and stability

Edge weights are the key to the closeness of the association between symptoms, so their estimation accuracy is crucial. To evaluate this accuracy, bootstrapping was employed to determine the 95% Confidence Intervals (CI) for edge weights using the R package *bootnet*. High accuracy is indicated if the original sample edge weights fall within the bootstrap CI. A stability test validated the robustness of each node’s centrality metrics, which are crucial for the network’s stability. The network was re-estimated using case-dropping subset bootstrap, and centrality stability was tested by correlating bootstrap and original network centrality rankings. Centrality indicators for EI and BEI were assessed using correlation stability (CS) coefficients, with values over 0.25 indicating moderate stability and over 0.5 indicating strong stability (23). In addition, we calculated differences in node centrality and edge weights using bootstrapping and constructed CIs. The test results are shown in **Supplementary Figures S1**, **S2**, **Appendix A**.

#### 2.3.4 Subgroup analysis

Given that age, gender, and education level may affect sleep quality and the psychological state of TB patients, we conducted subgroup analyses for these factors. For gender, we applied the Network Comparison Test (NCT) to assess the differences in global and local connectivity between male and female symptom networks. For age, we divided all subjects nearly equally into three groups: age group I (14-46 years, 206 participants), age group II (47-62 years, 203 participants), and age group III (63-92 years, 203 participants). The equal division into 3 age groups enabled each group to have a sufficient sample size and ensured the robustness of the network comparisons. NCTs in pairs (three times in total) were then conducted on the three age groups to analyze age differences between the symptom networks. For education, all participants were categorized into 3 levels: education level I (junior high school and below, 413 participants), education level II (secondary vocational school/senior high school, 101 participants), and education level III (junior college/undergraduate and above, 98 participants). Pairwise NCTs were still performed at all three education levels to analyze structural differences in symptom networks. NCTs of age, gender, and education were performed using the R package *NetworkComparisonTest* (version 2.2.2).

## 3 Results

### 3.1 Descriptive statistics

In total, 612 TB inpatients were enrolled (376 men and 236 women, mean age 52.95 ± 17.83 years). Among all participants, the detection rates of insomnia (PSQI>10), depression (PHQ-9 ≥ 10), anxiety (GAD-7 ≥ 10) were and 41.7%, 40.2%, 36.0%. In addition, the median stigma score (interquartile range (IQR)) was 11 (6, 13).

### 3.2 Network structure

#### 3.2.1 General structure

**Figure 1:**
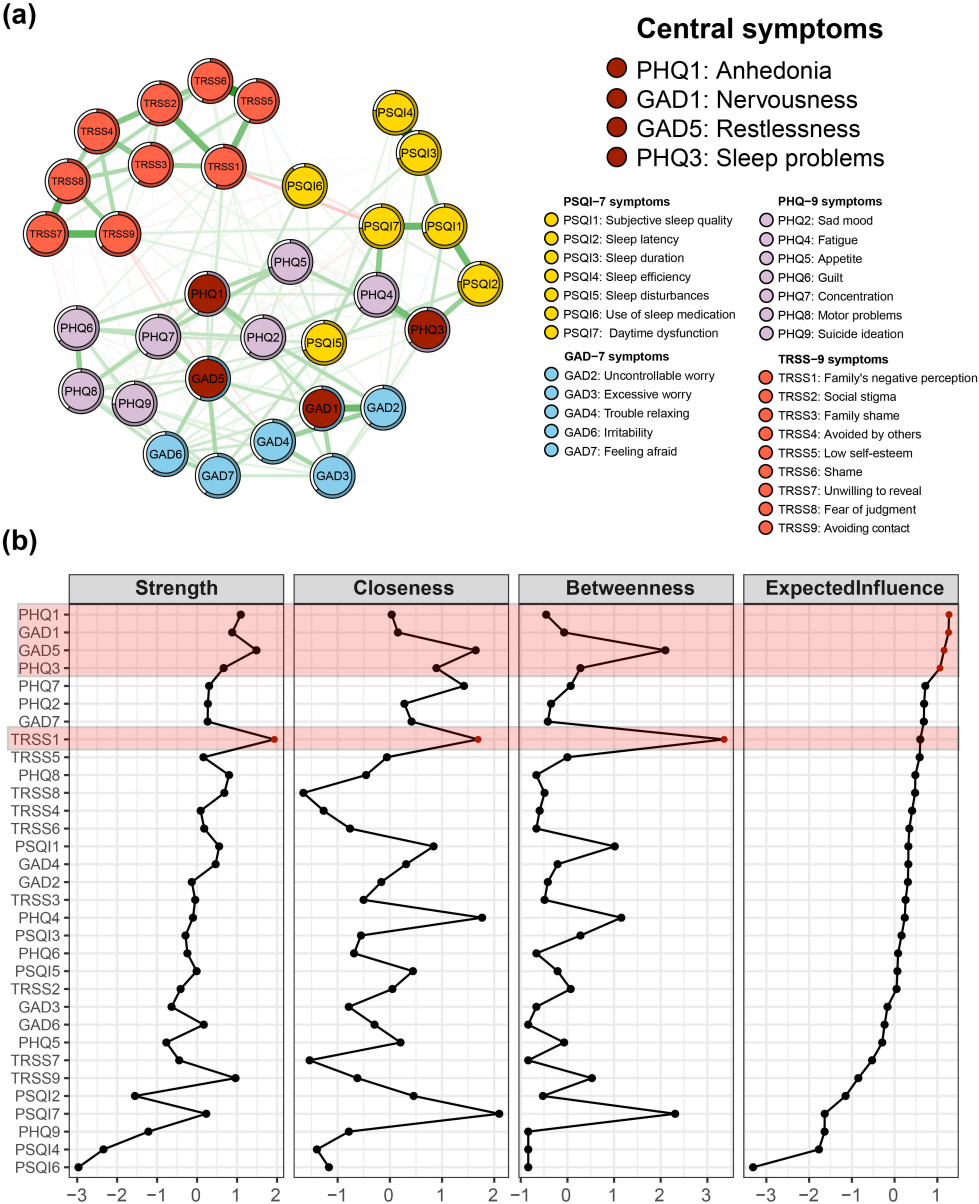
Central symptoms of insomnia-depression-anxiety-stigma in TB patients. **(A)** The symptom network structure of insomnia-depression-anxiety-stigma. Red circles indicate central symptoms, green lines indicate positive associations, and red lines indicate negative associations. **(B)** Centrality measures of symptom networks, including Strength Centrality, Closeness Centrality, Betweenness Centrality, and Expected Influence (EI).

**Figure 1A** shows the symptom network of insomnia, depression, anxiety, and stigma. The network consists of 32 nodes, partitioned into four communities: the insomnia community with 7 nodes, the depression community with 9 nodes, the anxiety community with 7 nodes, and the stigma community with 9 nodes. In total, there are 496 edges in the network, of which 193 (38.9%) were statistically significant (*P* < 0.05), indicating strong interconnectivity between the four categories of symptom communities.

#### 3.2.2 Strong edges

The 10 edges with the strongest partial correlations observed in the model were distributed within their specific symptom communities (i.e., 1 edge in the depression community, 1 edge in the anxiety community, 3 edges in the insomnia community, and 5 edges in the stigma community). The strongest correlation was observed between PSQI3 (sleep duration) and PSQI4 (sleep efficiency), yielding a Partial Correlation Coefficient (PCC) of 0.52.

#### 3.2.3 Predictability

The predictability of the nodes ranges from 56%-91%, with an average of 64.16%. This indicates that 64.16% of the variance in the nodes within the specified network can be explained by their neighboring nodes. Notably, PSQI6 (sleep medication) demonstrates the highest predictability, reaching 91%.

### 3.3 Network centrality

The centrality indicators of the overall symptom network structure are presented in **Figure 1B**, **Table 1**. A shift in the Expected Influence (EI) served as a critical basis for determining the central nodes within the network. In the EI panel of **Figure 1B**, a significant decline in EI occurred from PHQ3 to PHQ7, so the centrality symptoms of the entire network were designated as PHQ1 (anhedonia), GAD1 (nervousness), GAD5 (restlessness), and PHQ3(sleep problems). PHQ1 had the highest EI value of 1.27 and was in the closest center of the symptom network. GAD1 was second (EI = 1.26) and GAD5 was third (EI = 1.16). In fourth place was PHQ3 (EI = 1.06).

**Table 1:** Statistical description of measurement items.

Item abbreviation	Item content	Item median scores (IQR)	Expected influence	Predictability
PSQI1	Subjective sleep quality	1 (1, 2)	0.32	0.7
PSQI2	Sleep latency	2 (1, 2)	-1.15	0.77
PSQI3	Sleep duration	2 (1, 2)	0.16	0.71
PSQI4	Sleep efficiency	1 (0, 2)	-1.77	0.77
PSQI5	Sleep disturbances	1 (1, 2)	0.07	0.69
PSQI6	Sleep medication	0 (0, 1)	-3.31	0.91
PSQI7	Daytime dysfunction	1 (1, 2)	-1.63	0.74
GAD1	Nervousness	1 (1, 2)	1.26	0.56
GAD2	Uncontrollable worry	1 (0, 1)	0.31	0.61
GAD3	Excessive worry	1 (0, 1)	-0.17	0.63
GAD4	Trouble relaxing	1 (0, 1)	0.32	0.59
GAD5	Restlessness	1 (0, 2)	1.16	0.56
GAD6	Irritability	1 (0, 1)	-0.23	0.6
GAD7	Feeling afraid	0 (0, 1)	0.68	0.6
PHQ1	Anhedonia	1 (0, 2)	1.27	0.57
PHQ2	Sad mood	1 (0, 1)	0.69	0.61
PHQ3	Sleep problems	1 (0, 2)	1.06	0.65
PHQ4	Fatigue	1 (1, 2)	0.24	0.64
PHQ5	Appetite	1 (0, 2)	-0.29	0.71
PHQ6	Guilt	1 (0, 1)	0.08	0.65
PHQ7	Concentration	1 (0, 1)	0.72	0.59
PHQ8	Motor problems	0 (0, 1)	0.49	0.6
PHQ9	Suicide ideation	0 (0, 1)	-1.64	0.75
TRSS1	Family’s negative perception	1 (0, 2)	0.6	0.56
TRSS2	Social stigma	1 (0, 1)	0.05	0.58
TRSS3	Family shame	1 (0, 2)	0.26	0.6
TRSS4	Avoided by others	1 (0, 2)	0.41	0.6
TRSS5	Low self-esteem	1 (0, 2)	0.59	0.56
TRSS6	Shame	1 (0, 1)	0.35	0.56
TRSS7	Unwilling to reveal	1 (1, 2)	-0.53	0.63
TRSS8	Fear of judgment	1 (0, 2)	0.48	0.58
TRSS9	Avoiding contact	1 (0, 2)	-0.85	0.65

IQR, interquartile range.

EI of stigma was generally ranked lower in the symptom network. Although TRSS1 (family’s negative perception) had the highest EI within the stigma community, it was only ranked 8th overall in the network. However, it is noteworthy that the TRSS1 symptom had the highest mediator centrality, much higher than any other symptom (Betweenness panel of **Figure 1B**). Further observing the Strength panel and Closeness panel in **Figure 1B**, we found that TRSS1 ranked high in Strength Centrality and Closeness Centrality.

### 3.4 Bridge symptoms

**Figure 2:**
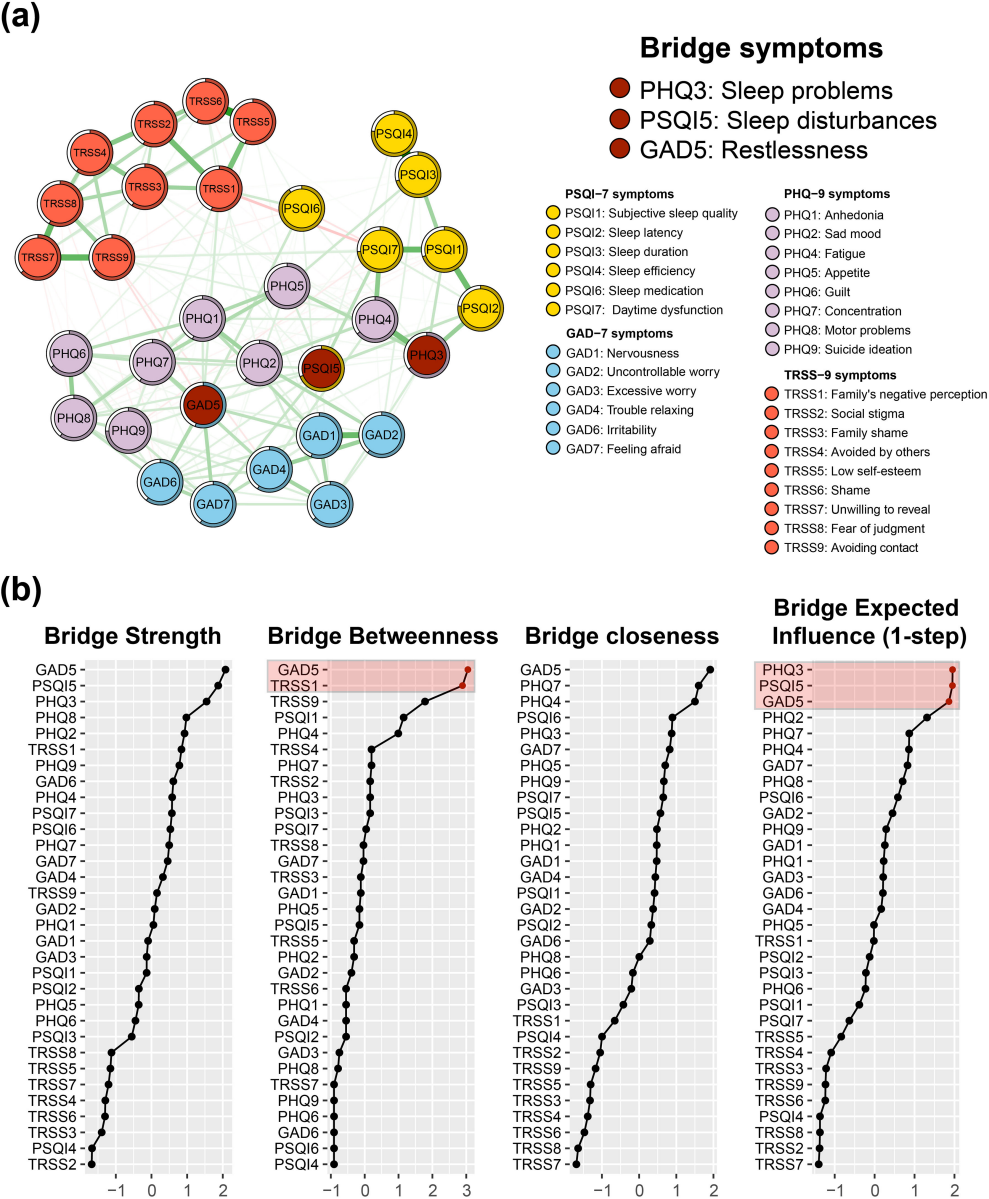
Bridge symptoms of insomnia-depression-anxiety-stigma in TB patients. **(A)** The symptom network structure of insomnia-depression-anxiety-stigma. Red circles indicate bridge symptoms, green lines indicate positive associations, and red lines indicate negative associations. **(B)** Bridge measures of symptom networks, including Bridge Strength Centrality, Bridge Closeness Centrality, Bridge Betweenness Centrality, and Bridge Expected Influence (BEI).

**Figure 2A** shows the Bridge Strength Centrality, Bridge Betweenness Centrality, Bridge Closeness Centrality, and BEI for the 32 nodes in the symptom network. According to the BEI panel in **Figure 2B**, the BEI value takes a steep drop between GAD5 and PHQ2, so the bridge symptoms of this network are designated as PHQ3 (sleep problems), PSQI5 (sleep disturbances), and GAD5 (restlessness).

The symptom item in the other community nearest to PHQ3 is PSQI2 (sleep latency), suggesting that the depression cluster may be primarily linked to the insomnia symptom cluster via PHQ3 - PSQI2. The closest item belonging to the other community with PSQI5 is PHQ2 (sad mood), suggesting that the insomnia symptom cluster may be linked to the depression symptom cluster primarily through PSQI5 - PHQ2. The closest item belonging to the other community with GAD5 is PHQ7 (concentration), suggesting that the anxiety symptom cluster may be primarily linked to the depression symptom cluster through GAD5 - PHQ7.

Focusing on the bridge-betweenness part of the whole network (**Figure 2B** Bridge Betweenness panel), we find that similar to the case of the centrality metrics, TRSS1 still has high intermediation properties, with the second highest bridge-betweenness value.

### 3.5 Accuracy and stability of the network

**Figure 3:**
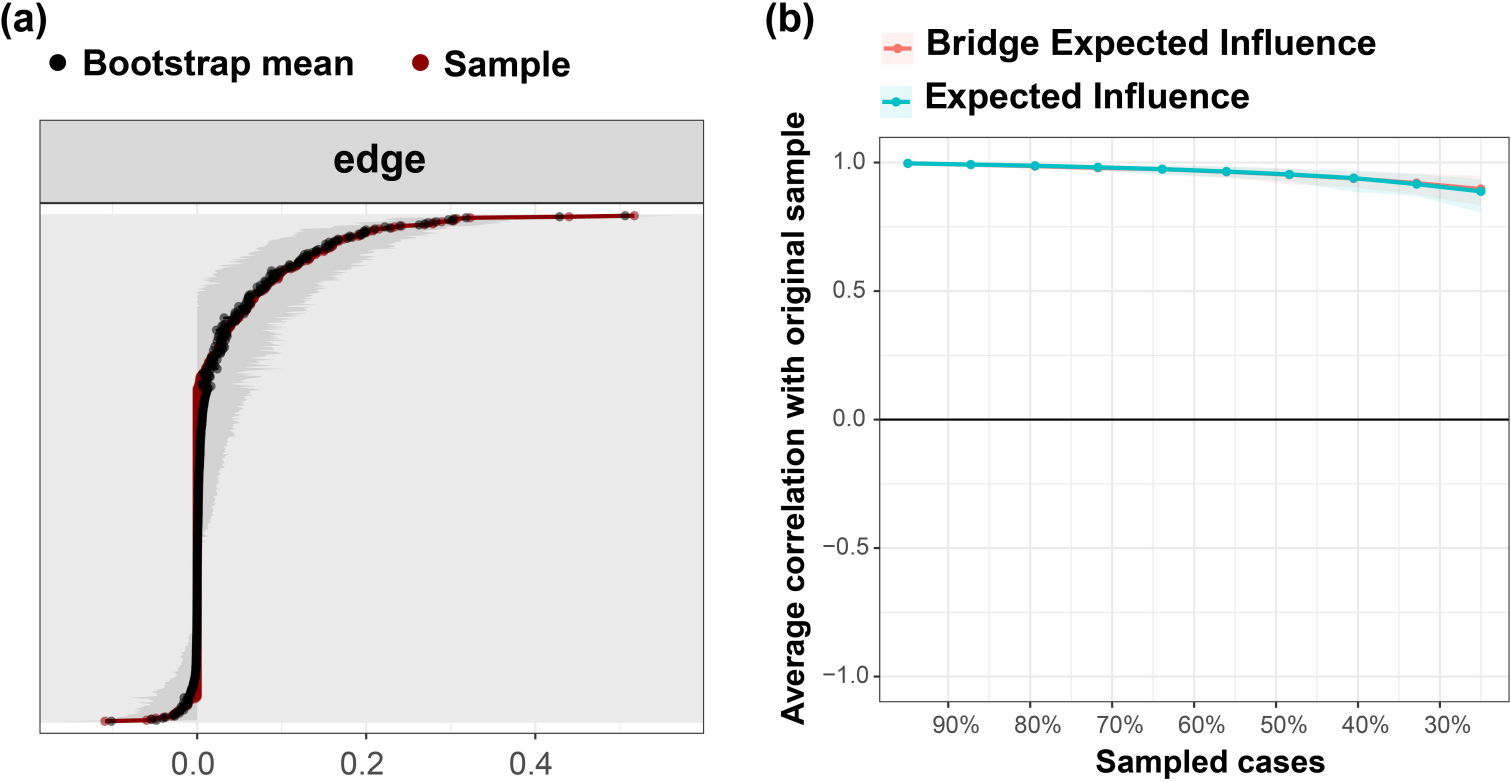
Accuracy and stability tests of the symptom network. **(A)** 95% confidence intervals for network edge weights derived from bootstrapping 1000 times. **(B)** Average correlation curve with the original sample derived from case-dropping subset bootstrap 1000 times. The green curve shows the correlation coefficient between the Expected Influence (EI) ranking after deleting a certain percentage of the sample and the ranking of the original sample. The red curve shows the correlation coefficient between the Bridge Expected Influence (BEI) ranking after deleting a certain percentage of samples and the ranking of the original samples.

**Figure 3A** shows the accuracy test for all the edge weights in the network. The 1000-times bootstrap means of all the edge weights largely coincide with the original sample results, and almost all sample points fall inside the 95% confidence intervals of the bootstrap results. This indicates that the edge weights of the networks constructed in this study have a high degree of accuracy, implying the stability of the edge or partial correlations in the networks.

**Figure 3B** shows the results of the central stability test of the symptomatic network. The CS coefficients of both the BEI and the EI calculated from the results of 1000-times bootstrap are both 0.75, which indicates that the network constructed in this study has a sizable degree of stability.

**Supplementary Figures S1**, **S2** illustrate the difference test for edge weights and node Strength Centrality. It can be observed that the statistical differences between the nodes become more significant as the Strength Centrality increases or decreases, with the middle-ranked nodes having the least significant statistical differences from the other nodes (**Supplementary Figure S1**). The statistical differences in edge weights have similar characteristics (**Supplementary Figure S2**). Overall, the majority of nodes and edges exhibit significant statistical differences, indicating that the network is robust.

### 3.6 Differences in network structure by gender, age, and education

**Supplementary Figure S3** illustrates the insomnia-depression-anxiety-stigma symptom networks for men and women. We performed a global strength invariance test for both networks. The global Strength Centrality was 15.18 and 15.13 for males and females, respectively, and the difference was not statistically significant (P > 0.1).

As for the differences in network structure between different age groups, none of the three age groups (age group I:14-46 years, age group II:47-62 years, and age group III:63-92 years) had statistically significant differences in network structure (**Supplementary Figure S4**). The P-values for two-by-two comparisons were 0.61 (age group I vs. age group II), 0.90 (age group I vs. age group III), and 0.66 (age group II vs. age group III).

The differences in network structure among the three education levels (education level I: junior high school and below, education level II: secondary vocational school/senior high school, and education level III: junior college/undergraduate and above were not statistically significant (**Supplementary Figure S5**). The P-values for pairwise NCTs were 0.44 (education level I vs. education level II), 0.74 (education level I vs. education level III), and 0.50 (education level II vs. education level III).

## 4 Discussion

For the first time, this study employed symptom network analysis to reveal the interactions between insomnia, depression, anxiety, and stigma among TB patients. Consistent with previous symptom network studies on mental disorders (24–26), we found that the strongest edges were all distributed within their respective communities, rather than between communities. The strong connections among nodes within each community suggest that the four scales utilized (PHQ-9, GAD-7, PSQI, and TRSS) exhibit excellent measurement effects. Notably, the most significant edge in the overall symptom network was PSQI3-PSQI4, highlighting a strong correlation between these two factors in TB patients, a finding supported by related studies (16, 25). Of interest, the stigma community comprised the largest percentage (50%) of the top ten edges in the overall symptom network, suggesting that this community exhibits the strongest degree of linkage across all symptoms. Thus, intervening in specific key symptoms in this community could significantly improve the stigma symptom cluster. Furthermore, our analysis revealed that age, gender, and education do not influence the symptom network structure of insomnia, depression, anxiety, and stigma in TB patients, as confirmed by network global invariance testing. This finding is also consistent with results from numerous studies (15, 25, 27).

The highest EI were detected for PHQ1 (anhedonia), GAD1 (nervousness), GAD5 (restlessness), and PHQ3 (sleep problems), identifying them as central symptoms within the network. This suggests that targeting these specific symptoms in treatment could be particularly effective for patients with TB. Among the central symptoms, PHQ1 occupies the most central position in the symptom network, indicating that anhedonia plays the most crucial role in the entire network. As a key symptom of depression, anhedonia is linked to worse outcomes like higher suicide risk and poor treatment response. This may be due to dysfunctions in the brain’s reward system or the mesolimbic dopaminergic system, which are increasingly recognized as fundamental characteristics of depressive disorders (28). Similarly, a large sample (n>2000) analysis of the schizophrenia-depression-anxiety-autism-obsessive compulsive disorder network revealed that anhedonia had the highest Strength Centrality across the network (29). Additionally, anhedonia is characteristic not only of depression but also of certain anxiety disorders and other psychiatric conditions, including substance abuse and schizophrenia (30). Hence, it is reasonable to hypothesize that intervening in TB patients’ anhedonia can alleviate the entire symptoms in the insomnia-depression-anxiety-stigma network. Unfortunately, effective pharmacological treatments specifically for anhedonia in depressed patients remain lacking (31). However, psychological interventions such as cognitive behavioral therapy (CBT), augmented depression therapy (ADepT), and Positive Affect Treatment (PAT) have shown some effectiveness in addressing this symptom (30, 32). Other central symptoms such as GAD1 (nervousness), GAD5 (restlessness), and PHQ3 (sleep problems) exhibited slightly lower EI values than PHQ1. Nevertheless, another network study of Macau residents during the COVID-19 pandemic showed GAD4 (trouble relaxing), GAD6 (irritability), and PHQ4 (fatigue) as central symptoms (25). This discrepancy may be attributed to differences in research subjects or the specific conditions being studied.

Identifying bridge symptoms within the network is crucial for understanding the development and maintenance of comorbidities, and helps clinicians to pinpoint relevant treatment targets. By calculating the BEI of the whole symptom network, this study identified PHQ3 (sleep problems), PSQI5 (sleep disturbances), and GAD5 (restlessness) as bridge symptoms, which were the most likely candidates for triggering or enhancing the other symptoms. Further, we identified two key bridges PSQI2(sleep latency)-PHQ3(sleep problems) and PSQI5(sleep disturbances)-PHQ2(sad mood) connecting the depression and insomnia clusters based on edge weights. Depression and insomnia often co-occur and share a bidirectional relationship (33). High-quality longitudinal data have established insomnia as an independent risk factor for depression onset (34–36). However, the specific items of depression and insomnia that are associated remain unclear (37). Our findings suggest a closer relationship between TB-related depression and insomnia, particularly the long sleep latency and sleep disturbances induced by the TB disease itself (e.g., cough and dyspnea). Hence, ‘sleep latency’ and ‘sleep disturbances’ emerged as critical priorities due to their connections with ‘sleep problems’ and ‘sad mood’ in depression. Targeting these two symptoms may represent an effective intervention strategy for alleviating both insomnia and depression in TB patients. Additionally, GAD5(restlessness)-PHQ7(concentration) was identified as a bridge linking the anxiety community and the depression community. Given that TB is a chronic wasting disease, its clinical signs—including prolonged respiratory impairments and the neurotoxic effects of anti-tuberculosis drugs—may contribute to symptoms of restlessness and inattention (38). Based on our findings, targeting the bridging symptoms PHQ3, PSQI5, and GAD5 in interventions may be effective in preventing the co-morbidity of insomnia, depression, and anxiety among TB patients.

Across the symptom network, while the EI and the BEI of the stigma community are both at low levels, it is worth noting that the Betweenness Centrality of node TRSS1 (“I feel that my family members look down on me for having tuberculosis”) is extremely high. This suggests that discrimination from the family has a critical mediating role in stigmatized communities and even in the entire symptom network. A structural-equation study of family functioning, doctor-patient communication, TB knowledge, and depression among TB patients found that stigma played a significant mediating role in the association between family functioning and depression, strongly supporting our results (21). Previous literature reported that family perceptions and acceptance of people with mental disorders play a crucial role in the course and outcome of the illness (39). Additionally, stigma has been shown to significantly impede recovery from mental disorders (40), while the implementation of self-stigma interventions has demonstrated promise in facilitating a quicker recovery from psychiatric symptoms (41). Furthermore, adverse family environments and discrimination can severely impact patients’ life satisfaction and social confidence, potentially exacerbating depression and suicidal tendencies (42). In line with our study, TB patients are generally highly stigmatized, with TRSS scores almost reaching a mean of 10. In summary, addressing the stigma of TB patients is essential, particularly by ensuring that these individuals receive adequate family support. Such support may provide extraordinary benefits for psychological well-being and TB disease recovery.

## 5 Limitations

Although this is the first network analysis of insomnia-depression-anxiety-stigma symptoms in TB patients, it has some limitations. First, it is a cross-sectional study, which does not lend itself to a causal explanation. Second, self-reported measures were used to assess insomnia and mental symptoms, which may be subject to recall bias and are limited in capturing clinical phenomena. Third, patient’s medication, first onset, duration of illness may limit the interpretation and generalization of our results to some extent.

## 6 Conclusion

This study employed symptom network analysis to illustrate the interactions between insomnia, depression, anxiety, and stigma in TB patients. We found that depression (anhedonia and sleep problems) and anxiety (nervousness and restlessness) were central symptoms in the symptom network. Additionally, a total of three bridge symptoms were identified: PHQ3 (sleep problems), PSQI5 (sleep disturbances), and GAD5 (restlessness). Notably, this study is the first to highlight the significant mediating role of stigma, particularly family’s negative perception, in the maintenance of insomnia and psychological symptom networks in TB patients. Consequently, it is critical that treatment approaches not only address the central and bridging symptoms associated with insomnia, depression, and anxiety but also include strategies to mitigate family-related stigma. Targeting these symptoms may enhance therapeutic outcomes and improve the sleep quality and mental health of TB patients.

## Data Availability

The raw data supporting the conclusions of this article will be made available by the authors, without undue reservation.
