# Supplementary material for: Symptom network analysis of insomnia-depression-anxiety-stigma in tuberculosis patients
Source: Front Psychiatry. 2025 Jan 23;15:1513524. doi: 10.3389/fpsyt.2024.1513524 (PMC11798919; doi:10.3389/fpsyt.2024.1513524)
Supplement: Supplementary file 1 [file DataSheet1.docx]

Supplementary Material

**Symptom network analysis of insomnia-depression-anxiety-stigma in tuberculosis patients**

**Xiangmin Liu^1^, Xue Qiu^1^****, Huizhen Lan^2^, LiuYue Diao^1^, Wei Huang^3^, Yan Wen^1^,**

**Mei Feng^1, 4*^, Xiangdong Tang^5*^**

^1^ Department of Pulmonary and Critical Care Medicine, West China Hospital, Sichuan University /West China School of Nursing, Sichuan University, Chengdu, China

^2^ Department of Intensive Care Unit, The Fourth People Hospital of Nanning, Nanning, China

^3^ Chengdu Center for Disease Control and Prevention, Chengdu, China

^4^ Nursing Key Laboratory of Sichuan Province, Chengdu, China

^5^ Mental Health Center/Neurobiology Monitoring Center, West China Hospital, Chengdu, China

* Corresponding to:

Mei Feng [(fm197711@163.com)](mailto:(fm197711@163.com)) and

Xiangdong Tang, M.D., Ph.D. ( E-mail: [2372564613@qq.com](mailto:2372564613@qq.com) )

Professor Tang, Mental Health Center, West China Hospital, Sichuan University

No.28 Dian Xin Nan Jie, Chengdu, Sichuan, China

Phone:86-28-85422733;Fax:86-28-85422632; https://orcid.org/0000-0001-6876-3328

# Supplementary Figures and Tables

## Supplementary Figures


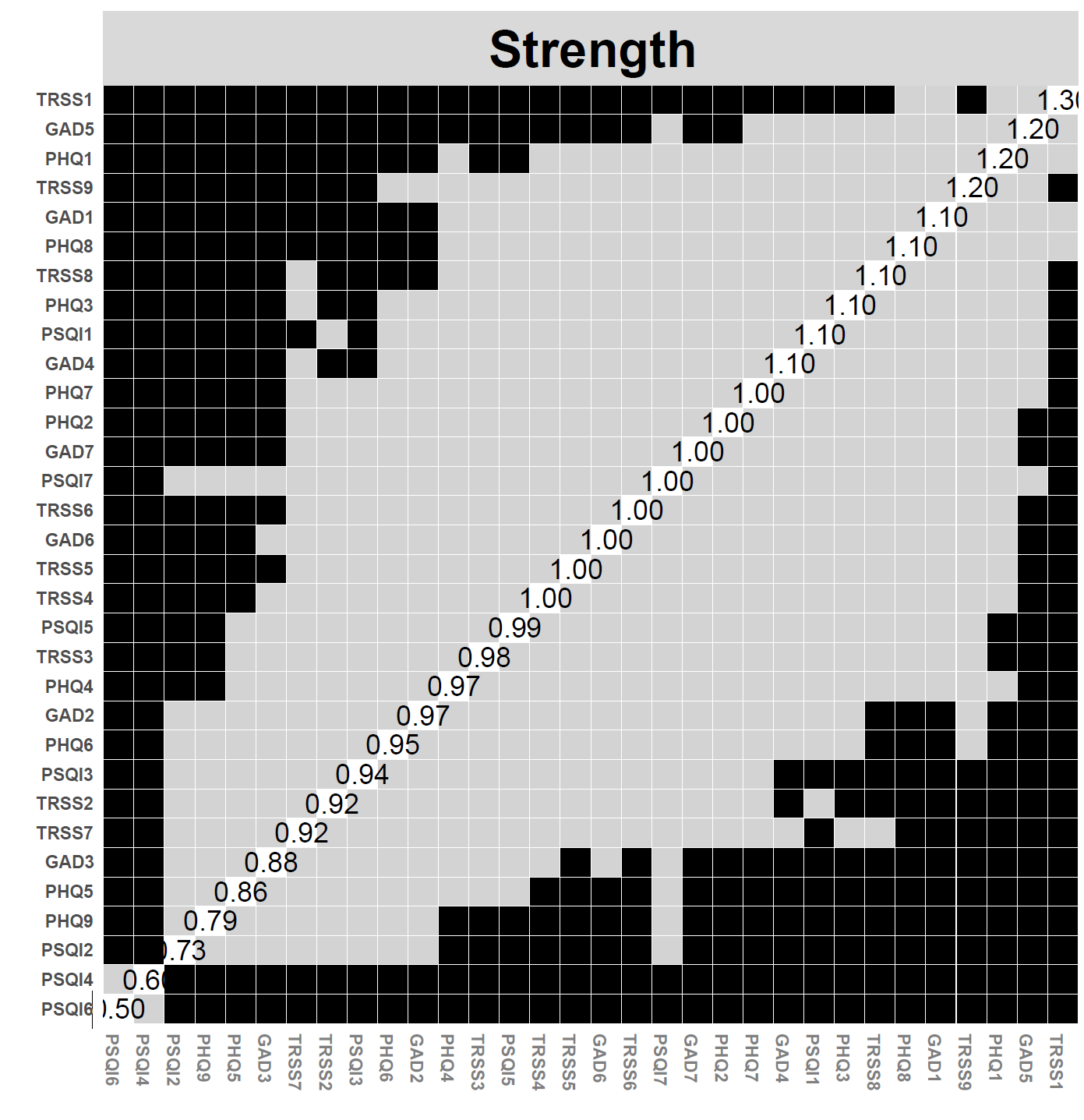


**Figure S1. Test for Difference in Nodal Strength Centrality.**

Gray boxes represent that the strength centrality of the two corresponding nodes is not statistically different while black boxes represent that it is statistically different (*P* < 0.05). Values on the main diagonal of the matrix indicate the strength centrality of the corresponding node.


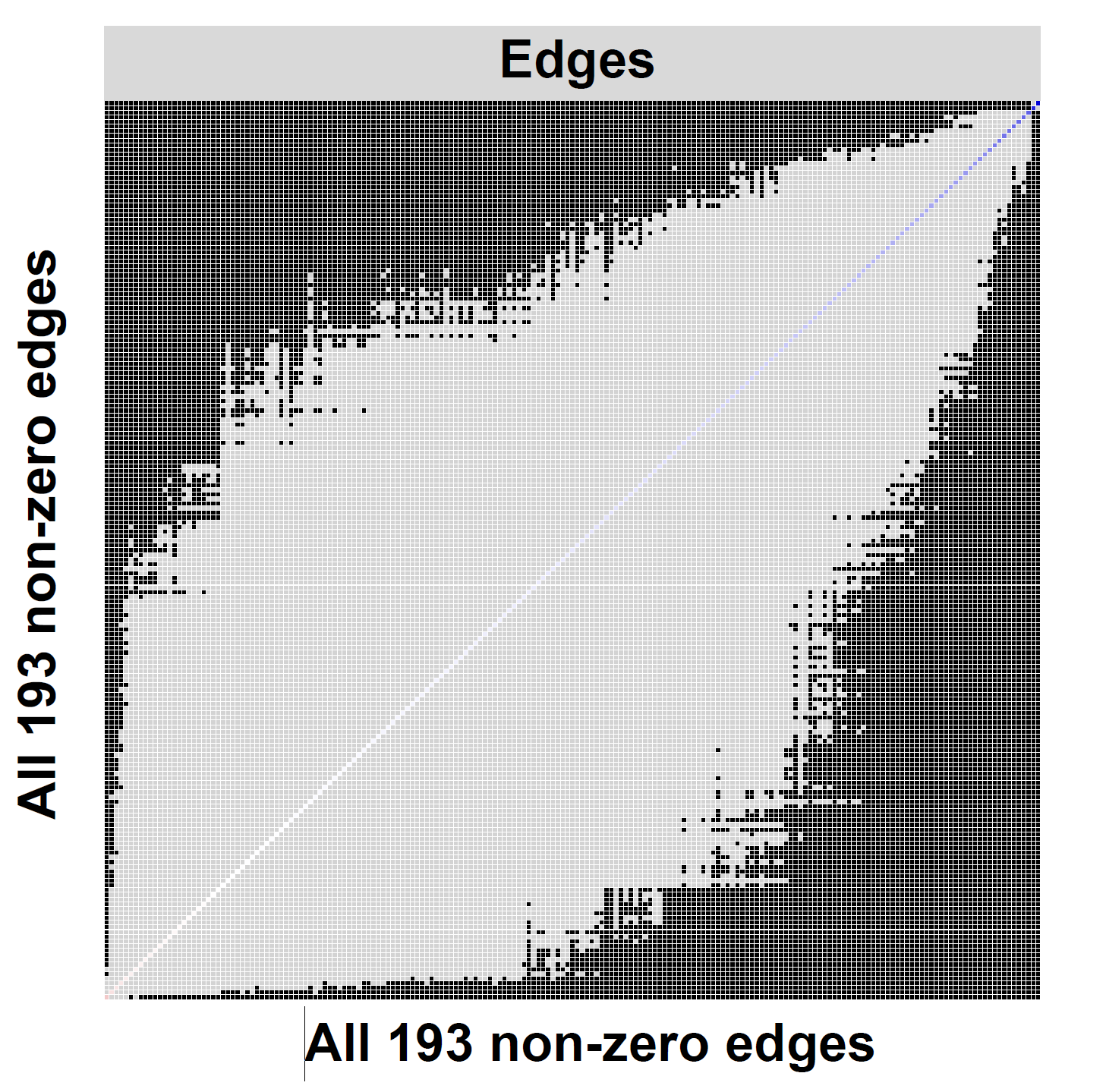


**Figure S2. Test for Difference in Edge Weights.**

Gray boxes represent two corresponding edge weights that are not statistically different and black boxes represent two corresponding edge weights that are statistically different (*P* < 0.05). The colored boxes on the main diagonal represent the color of the edge weights in the variable network. The blue color represents a positive relationship and the red color represents a negative relationship.


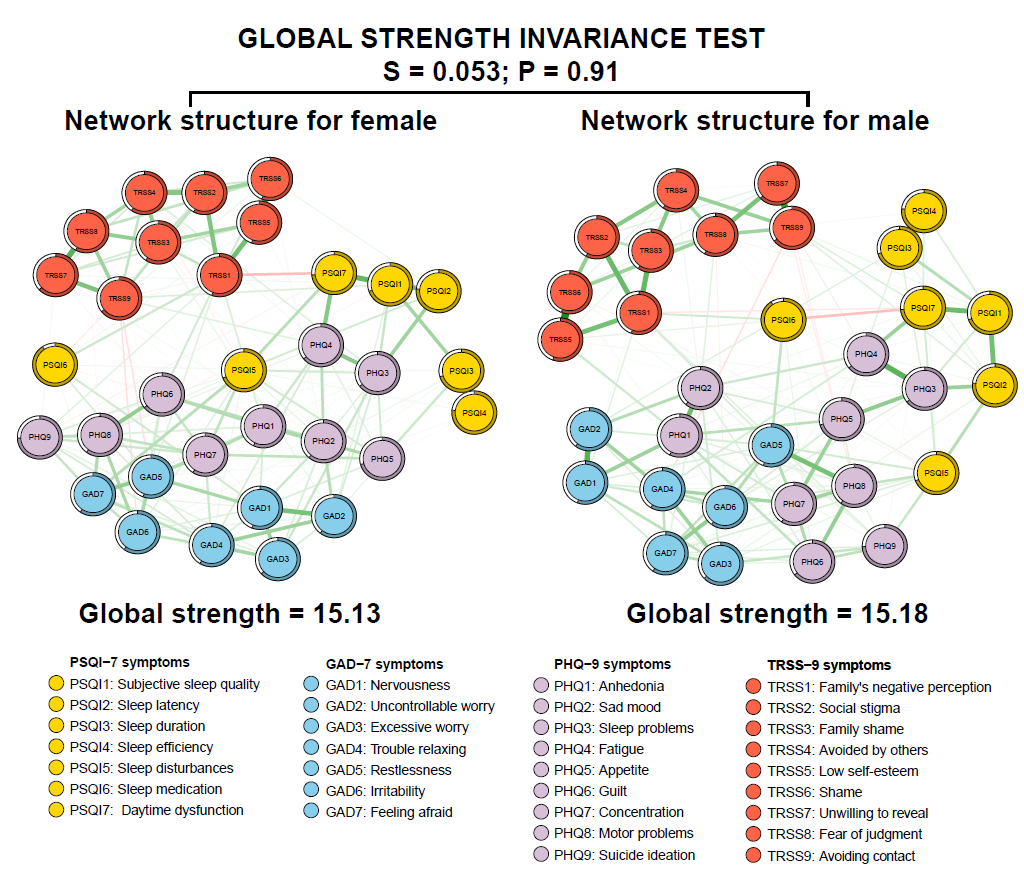


**Figure S3. Comparison of symptom networks in male and female TB patients.**

The Network Comparison Test (NCT) was applied to assess differences in global and local connectivity between male and female symptom networks.

There were no statistically significant differences (*P* = 0.91) in the structure of the insomnia-depression-anxiety-shame symptom network between males and females.


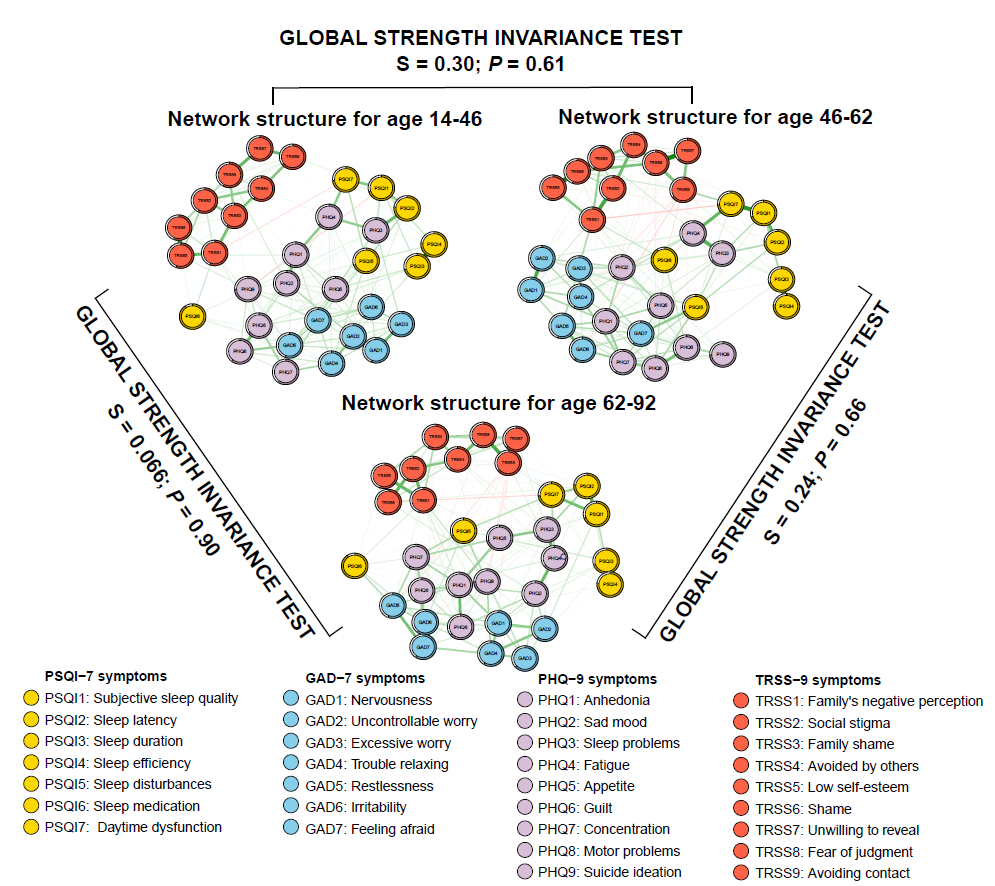


**Figure S4. Comparison of symptom networks** **between** **different age groups.**

The Network Comparison Test (NCT) was applied to assess differences in global and local connectivity between three groups: age group I (14-46 years, 206 participants), age group II (47-62 years, 203 participants), and age group III (63-92 years, 203 participants).

There were no statistically significant differences (*P* > 0.1) in the structure of the insomnia-depression-anxiety-shame symptom network between the three age groups.


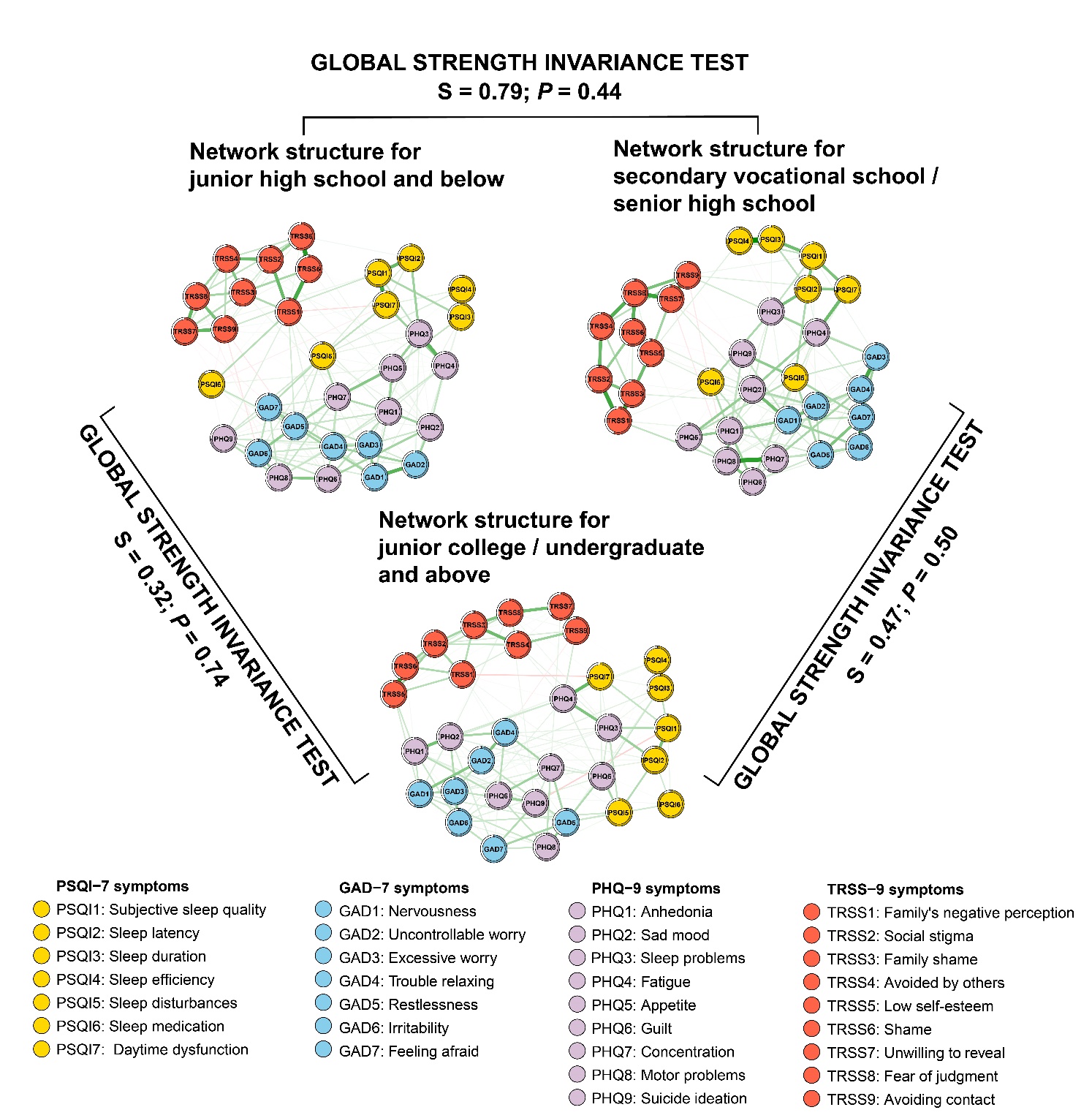


**Figure S5. Comparison of symptom networks** **between different** **education levels.**

The Network Comparison Test (NCT) was applied to assess differences in global and local connectivity between three groups: education level I (junior high school and below, 413 participants), education level II (secondary vocational school / senior high school, 101 participants), and education level III (junior college / undergraduate and above, 98 participants).

There were no statistically significant differences (*P* > 0.1) in the structure of the insomnia-depression-anxiety-shame symptom network between the three education levels.

## Supplementary Tables

**Table S1. Abbreviation, content and description of all scale items.**

| Item abbreviation | Item content | Item description |
| --- | --- | --- |
| PSQI1 | Subjective sleep quality | subjective sleep quality |
| PSQI2 | Sleep latency | sleep latency |
| PSQI3 | Sleep duration | sleep duration |
| PSQI4 | Sleep efficiency | sleep efficiency |
| PSQI5 | Sleep disturbances | sleep disturbances |
| PSQI6 | Sleep medication | use of sleeping medication |
| PSQI7 | Daytime dysfunction | daytime dysfunction |
| GAD1 | Nervousness | Feeling nervous, anxious or on edge |
| GAD2 | Uncontrollable worry | Not being able to stop or control worrying |
| GAD3 | Excessive worry | Worrying too much about different things |
| GAD4 | Trouble relaxing | Trouble relaxing |
| GAD5 | Restlessness | Being so restless that it is hard to sit still |
| GAD6 | Irritability | Becoming easily annoyed or irritable |
| GAD7 | Feeling afraid | Feeling afraid as if something awful might happen |
| PHQ1 | Anhedonia | Little interest or pleasure in doing things? |
| PHQ2 | Sad mood | Feeling down, depressed, or hopeless? |
| PHQ3 | Sleep problems | Trouble falling or staying asleep, or sleeping too much? |
| PHQ4 | Fatigue | Feeling tired or having little energy?. |
| PHQ5 | Appetite | Poor appetite or overeating? |
| PHQ6 | Guilt | Feeling bad about yourself—or that you are a failure or have let yourself or your family down? |
| PHQ7 | Concentration | Trouble concentrating on things, such as reading the newspaper or watching television? |
| PHQ8 | Motor problems | Moving or speaking so slowly that other people could have noticed? Or the opposite-being so fidgety or restless that you have been moving around a lot more than usual? |
| PHQ9 | Suicide ideation | Thoughts that you would be better off dead or of hurting yourself in some way? |
| TRSS1 | Family's negative perception | I feel that my family members look down on me for having tuberculosis |
| TRSS2 | Social stigma | I feel that my friends or neighbours look down on me for having tuberculosis |
| TRSS3 | Family shame | I feel that my friends or neighbours look down on my family because I have tuberculosis |
| TRSS4 | Avoided by others | I feel that my friends or neighbours avoid me on purpose |
| TRSS5 | Low self-esteem | I think less of myself for having tuberculosis |
| TRSS6 | Shame | I feel ashamed of myself for having tuberculosis |
| TRSS7 | Unwilling to reveal | I am unwilling to reveal my tuberculosis to my friends or neighbours |
| TRSS8 | Fear of judgment | I am afraid of going to TB clinics because other people may see me there |
| TRSS9 | Avoiding contact | I avoid keeping in touch with my friends or neighbours since I have tuberculosis |
